# Traumatic Pneumonia and Traumatic Tuberculosis

**Published:** 1917-04

**Authors:** 


**Traumatic Pneumonia and Traumatic Tuberculosis.** By F. PARKER WEBER, M.A., M.D., F.R.C.P. Lond. London: Adlard & Son. 1916. Price 6d.

This reprint from the *Clincial Journal* (July, 1916) directs attention to a subject of much interest and importance, from its connection with the difficult subject of compensation for accidents. It is too much the rule of judges and juries to ignore the traumatic element in cases of pneumonia and in heart cases. On the other hand, the writer was called upon some years ago to give evidence in court to the effect that a fatal pneumonia was not due to an accident, because it developed within a few hours of the injury ; the judge, however, gave a verdict in favour of the widow claiming compensation, on the ground that "when in doubt, give the widow the benefit thereof."

